# Angiotensin-Converting Enzyme 2 (ACE2), Transmembrane Peptidase Serine 2 (TMPRSS2), and Furin Expression Increases in the Lungs of Patients with Idiopathic Pulmonary Fibrosis (IPF) and Lymphangioleiomyomatosis (LAM): Implications for SARS-CoV-2 (COVID-19) Infections

**DOI:** 10.3390/jcm11030777

**Published:** 2022-01-31

**Authors:** Wenying Lu, Mathew Suji Eapen, Gurpreet Kaur Singhera, James Markos, Greg Haug, Collin Chia, Josie Larby, Samuel James Brake, Glen P. Westall, Jade Jaffar, Rama Satyanarayana Raju Kalidhindi, Nimesha De Fonseka, Venkatachalem Sathish, Tillie L. Hackett, Sukhwinder Singh Sohal

**Affiliations:** 1 Respiratory Translational Research Group, Department of Laboratory Medicine, School of Health Sciences, College of Health and Medicine, University of Tasmania, Launceston, TAS 7248, Australia; wenying.lu@utas.edu.au (W.L.); mathew.eapen@utas.edu.au (M.S.E.); jamesmarkos@bigpond.com (J.M.); greghaug@gmail.com (G.H.); collin.chia@ths.tas.gov.au (C.C.); josie.larby@ths.tas.gov.au (J.L.); sjbrake@utas.edu.au (S.J.B.); 2 National Health and Medical Research Council (NHMRC) Centre of Research Excellence (CRE) in Pulmonary Fibrosis, Respiratory Medicine and Sleep Unit, Royal Prince Alfred Hospital, Camperdown, NSW 2050, Australia; 3 Department of Anesthesiology, Pharmacology & Therapeutics, University of British Columbia, Vancouver, BC V6Z 1Y6, Canada; Gurpreet.Singhera@hli.ubc.ca (G.K.S.); tillie.hackett@hli.ubc.ca (T.L.H.); 4 UBC Centre for Heart Lung Innovation, St. Paul’s Hospital, Vancouver, BC V6Z 1Y6, Canada; 5 Department of Respiratory Medicine, Launceston General Hospital, Launceston, TAS 7250, Australia; 6 Department of Allergy, Immunology and Respiratory Medicine, The Alfred Hospital, Melbourne, VIC 3004, Australia; G.Westall@alfred.org.au (G.P.W.); jade.jaffar@monash.edu (J.J.); 7 Department of Immunology and Pathology, Monash University, Melbourne, VIC 3800, Australia; 8 Department of Pharmaceutical Sciences, School of Pharmacy, College of Health Professions, North Dakota State University, Fargo, ND 58105, USA; ram.kalidhindi@nih.gov (R.S.R.K.); nimesha.defonseka@ndsu.edu (N.D.F.); s.venkatachalem@ndsu.edu (V.S.)

**Keywords:** ACE2, TMPRSS2, Furin, IPF, LAM, COVID-19

## Abstract

We previously reported higher ACE2 levels in smokers and patients with COPD. The current study investigates if patients with interstitial lung diseases (ILDs) such as IPF and LAM have elevated ACE2, TMPRSS2, and Furin levels, increasing their risk for SARS-CoV-2 infection and development of COVID-19. Surgically resected lung tissue from IPF, LAM patients, and healthy controls (HC) was immunostained for ACE2, TMPRSS2, and Furin. Percentage ACE2, TMPRSS2, and Furin expression was measured in small airway epithelium (SAE) and alveolar areas using computer-assisted Image-Pro Plus 7.0 software. IPF and LAM tissue was also immunostained for myofibroblast marker α-smooth muscle actin (α-SMA) and growth factor transforming growth factor beta1 (TGF-β1). Compared to HC, ACE2, TMPRSS2 and Furin expression were significantly upregulated in the SAE of IPF (*p* < 0.01) and LAM (*p* < 0.001) patients, and in the alveolar areas of IPF (*p* < 0.001) and LAM (*p* < 0.01). There was a significant positive correlation between smoking history and ACE2 expression in the IPF cohort for SAE (r = 0.812, *p* < 0.05) and alveolar areas (r = 0.941, *p* < 0.01). This, to our knowledge, is the first study to compare ACE2, TMPRSS2, and Furin expression in patients with IPF and LAM compared to HC. Descriptive images show that α-SMA and TGF-β1 increase in the IPF and LAM tissue. Our data suggests that patients with ILDs are at a higher risk of developing severe COVID-19 infection and post-COVID-19 interstitial pulmonary fibrosis. Growth factors secreted by the myofibroblasts, and surrounding tissue could further affect COVID-19 adhesion proteins/cofactors and post-COVID-19 interstitial pulmonary fibrosis. Smoking seems to be the major driving factor in patients with IPF.

## 1. Introduction

The coronavirus disease 2019 (COVID-19) outbreak has caused more than 240 million infections so far with approximately 4.9 million deaths worldwide until October 2021 [1]. Chronic lung disease has been recognized as a risk factor for COVID-19 [2,3]. Smoking is one of the significant risk factors in chronic lung diseases such as chronic obstructive pulmonary disease (COPD) and lung cancer. A recent study has indicated that smokers have almost two-times higher odds of progression of COVID-19 severity than non-smokers, which confirms that smoking is a risk factor for COVID-19 progression [4]. We and others have previously reported increased expression of the severe acute respiratory syndrome-coronavirus-2 (SARS-CoV-2) receptor, angiotensin-converting enzyme 2 (ACE2) in smokers and patients with COPD [5,6]. Leung et al. [6] showed increased ACE2 gene expression in small airway epithelial cells of smokers and COPD patients, and the smoking status was significantly associated with the ACE2 gene expression levels in smokers, which was higher in smokers than non-smokers. Our study further indicated ACE2 protein expression was significantly increased in small airway epithelium, alveolar type II pneumocytes, and alveolar macrophages in current smokers with COPD compared to normal lung function smokers and normal controls [5]. We also reported that increased ACE2 expression was associated with increased endocytic vacuoles in smokers and COPD patients [7]. These results provided further evidence that smokers and COPD patients are highly susceptible to SARS-CoV-2 infection.

High expression of ACE2 is also a potential risk factor for severe COVID-19 outcomes in patients with interstitial lung diseases (ILD) [8]. Among the ILDs, idiopathic pulmonary fibrosis (IPF) is an irreversible fatal fibrotic lung disease with unknown etiology [9]. IPF affects over 3 million people worldwide and brings on a significant burden to patients, their families, and the healthcare system [10,11]. Risk factors including cigarette smoking, environmental exposures, microbial pathogens, and genetic factors play potential roles in IPF pathogenesis, which lead to alveolar epithelial cell injuries and replace injured alveolar epithelium with fibrotic tissue [12]. Patients with IPF usually have 2–4 years of survival after diagnosis and the mortality rate of IPF surpasses many types of cancers [8,13]. Another rare ILD mainly affecting females is lymphangioleiomyomatosis (LAM). LAM is a chronic and progressive rare neoplastic disease [14] that can occur sporadically (S-LAM) or associated with tuberous sclerosis (TS-LAM) [15,16]. LAM is characterized by hyperproliferation of abnormal mesenchymal “smooth muscle” like cells, resulting in multiple cysts and cystic lesions [17,18], which grow anomalously in the airway, parenchyma, axial lymphatics, and pulmonary blood vessels [19]. Research has found that a significant proportion of LAM patients shows inflammation in airways surrounded by LAM cells [20]. Histological observation shows LAM cysts lined with hyperplasic type II pneumocytes, possibly progressing through underlying chronic inflammation and fibrosis [20]. In both IPF and LAM diseases, the lung has prolonged airway losses, especially in distal parts of the lung parenchyma, leading to severe respiratory failure [21].

During the COVID-19 pandemic, patients with ILDs, such as IPF and LAM who had severe COVID-19 infection, experienced severe and fatal complications [22,23]. Therefore, understanding the role of the COVID-19-related biomarkers in ILDs would help to mediate and improve these patients’ treatment management. Apart from ACE2, Furin and Transmembrane protease serine 2 (TMPRSS2) are two additional proteins that help establish the SARS-CoV-2 virus infection [24]. ACE2, Furin and TMPRSS2 together play a pivotal role in COVID-19 pathophysiology [25,26]. Here, we examine the expression of ACE2, Furin, and TMPRSS2 in the lung parenchyma, especially in the small airway epithelium and alveolar regions of patients with IPF and LAM compared to normal healthy controls. We further evaluated the growth factor transforming growth factor beta1 (TGF-β1) and myofibroblast marker α-smooth muscle actin (α-SMA) in similar areas as with COVID-19 markers.

## 2. Materials and Methods

### 2.1. Subject Demographics

We used surgically resected lung tissue from healthy controls (HC, *n* = 12) from the James Hogg Lung Registry (Ethics ID: H00-50110), patients with end-stage IPF (*n* = 6) from the Alfred Health (Ethics ID: 336-13) and patients with LAM (*n* = 6) undergoing lung transplant provided from the National Disease Research Interchange (NDRI) Protocol DVES2 (with IRB exemption, North Dakota State University) (Table 1). All IPF patients were ex-smokers (mean ± SD: 18.2 ± 23.06), whereas LAM patients were never-smokers except one ex-smoker. In addition, HC tissues were provided from patients who had died of a cause other than respiratory diseases.

### 2.2. Immunohistochemistry (IHC) Staining

Lung tissue sections (3 µm) were cut from paraffin-embedded blocks individually. Tissue sections were treated with target retrieval solution (pH6, S169984, Dako, Victoria, Australia) in a Decloaking chamber at 110 °C for 15 min, followed by 3% hydrogen peroxide in water (*v*/*v*) (H1009, Sigma-Aldrich, Melbourne, Australia) for 15 min and with protein block solution (serum-free, X090930, Dako, Mulgrave, Australia) for 30 min at ambient temperature before applying the primary antibodies. The sections then were immunohistochemically stained using primary antibodies: ACE2 (1:800 dilution, ab15348, Abcam, Melbourne, Australia), TMPRSS2 (1:250 dilution, bs-6285R, Bioss antibodies, Redfern, Australia), Furin (1:200 dilution, bs-13228R, Bioss antibodies, Redfern, Australia), α-SMA (1:500, M0851, Dako, Mulgrave, Australia) and TGF-β1 (1:2000, ab215715, Abcam, Melbourne, Australia). The tissue slides were incubated in an IHC humidity chamber for 1 h at ambient temperature, followed by peroxidase-conjugated polymer backbone-carried secondary antibodies and visualized by 3,3′-diaminobenzidine (DAB) staining (EnVision™ Detection Systems, Peroxidase/DAB, Dako, Mulgrave, Australia).

### 2.3. Quantification of Staining

Images were obtained using a Leica camera ICC50 W and DM500 microscope (Leica Biosystem, Mount Waverley, Australia). The quantification of ACE2, TMPRSS2 and Furin positive expression in small airway epithelium and alveolar regions was carried through imaging software Image-Pro Plus 7.0 (Media Cybernetics, Rockville, MD, USA). Images of small airway epithelium and alveolar regions were taken as many as possible to cover the whole tissue section; overlapping of tissue areas were strictly avoided. Further, a minimum of five images of each subject’s small airway epithelium and alveolar regions were randomly selected using excel random number selection formula for the quantification measurement. All measurements were carried out using tissue imaging software Image-Pro Plus 7.0 and data presented here as the percentage of ACE2, TMPRSS2, and Furin expression in the small airway and alveolar regions.

### 2.4. Statistical Analysis

Following normality check, all data represented here were analyzed using non-parametric Kruskal–Wallis analysis of variance followed by multiple group comparisons Dunn’s test. Further, correlation analysis was performed with nonparametric Spearman correlation using GraphPad Prism V9.2, *p* < 0.05 is considered significant.

## 3. Results

### 3.1. Increase in ACE2, TMPRSS2 and Furin in IPF and LAM Lungs

**Figure 1:**
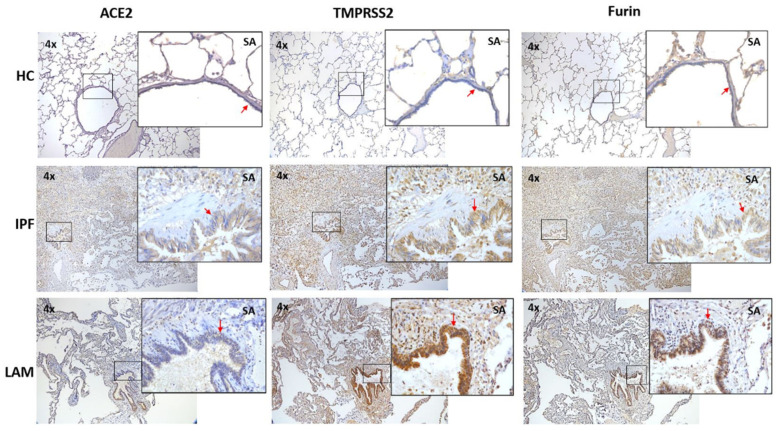
ACE2, TMPRSS2, and Furin expression on small airway epithelium and alveolar areas of IPF and LAM patients compared to HC. Representative images (4×) and inset images (40×) were taken using a bright field microscope. Each image from each group was taken from the same subject and the same area within the tissue. SA—small airways. Red arrows indicate the small airway epithelium.

**Figure 2:**
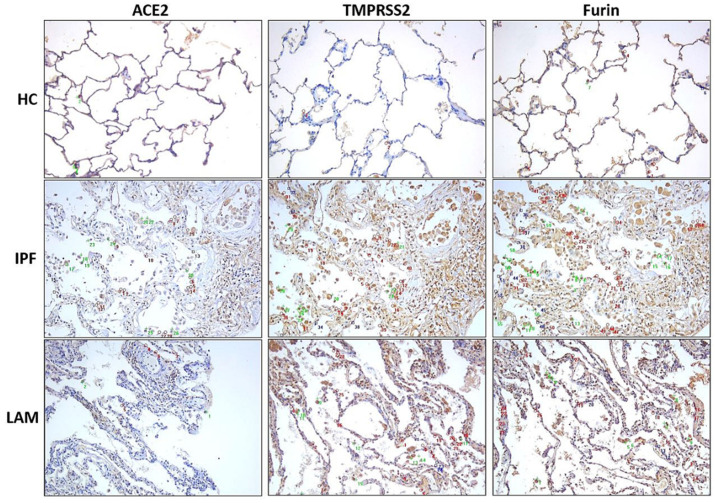
ACE2, TMPRSS2 and Furin expression in alveolar type II pneumocytes and macrophages. Red circles indicate type II pneumocytes; blue squares indicate type I pneumocytes; green crosses indicate alveolar macrophages. Representative images (20×) were taken using a bright field microscope. The cell type indication was processed with Image-pro Plus 7.0 software.

Overall, increased remodeling changes were observed in small airways and alveolar septal thickening in both IPF and LAM tissues, albeit higher expression in IPF patients than in LAM and HC tissues (Figure 1). We observed a concomitant upregulation of ACE2, TMPRSS2, and Furin expression on the SA epithelium and alveolar areas of IPF and LAM patients (Figure 1). We further observed that ACE2, TMPRSS2, and Furin expression was increased majorly in alveolar type II pneumocytes in IPF and LAM, especially in IPF patients, compared to HC (Figure 2). We also found the increased expression of these markers in alveolar macrophages in IPF and LAM patients (Figure 2).

**Figure 3:**
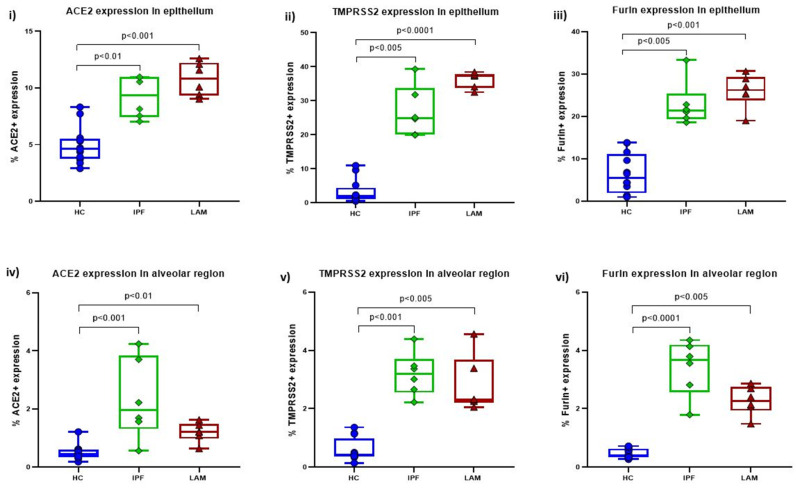
Quantitative analysis of (**i**) ACE2, (**ii**) TMPRSS2, and (**iii**) Furin expression in the small airway epithelium of IPF and LAM patients compared to HC. Quantitative analysis of (**iv**) ACE2, (**v**) TMPRSS2, and (**vi**) Furin expression in alveolar areas of IPF and LAM patients compared to HC. Significance is considered *p* < 0.05.

Compared to HC lung tissue (ACE2 median 4.65, range 2.89–8.32; TMPRSS2 median 1.98, range 0.47–10.94; Furin median 5.43, range 0.98–13.84), ACE2, TMPRSS2, and Furin expression were significantly upregulated in the airway epithelium of SAs of IPF (ACE2 median 9.35, range 7.05–10.98; TMPRSS2 median 24.88, range 19.9–39.28; Furin median 21.35, range 18.6–33.3; (*p* < 0.01)) and LAM (ACE2 median 10.84, range 9.02–12.61; TMPRSS2 median 37.37, range 32.52–38.41; Furin median 26.21, range 19.01–30.67; (*p* < 0.001)) patients (Figure 3i–iii). We also observed significant upregulation of ACE2, TMPRSS2, and Furin expression in the alveolar epithelium within the parenchyma of IPF (ACE2 median 1.95, rang 0.56–4.24; TMPRSS2 median 3.19, range 2.22–4.39; Furin median 3.68, range 1.79–4.35; (*p* < 0.001)) and LAM (ACE2 median 1.22, range 0.64–1.62; TMPRSS2 median 2.31, range 2.05–4.56; Furin median 2.27, range 1.48–2.87; (*p* < 0.01)) patients compared to HC (ACE2 median 0.44. rang 0.18–1.22; TMPRSS2 median 0.43, range 0.14–1.36; Furin median 0.41, range 0.27–0.72) (Figure 3iv–vi). These results match our previous findings with increased ACE2 expression in smokers and COPD [7].

**Figure 4:**
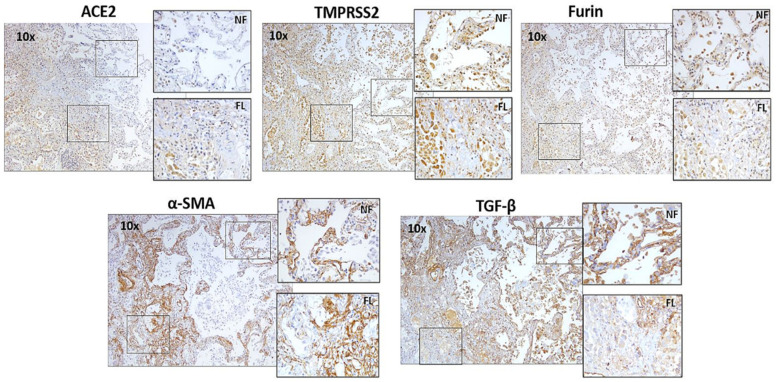
ACE2, TMPRSS2, Furin, myofibroblasts marker α-SMA, and growth factor TGF-β1 expression in fibrotic lesions and non-fibrotic lesions in IPF patients. Representative images (10×) and inset images (40×) were taken using a bright field microscope. Each marker image was taken from the same subject in the same tissue area. NF—non fibrotic lesion; FL—fibrotic lesion.

**Figure 5:**
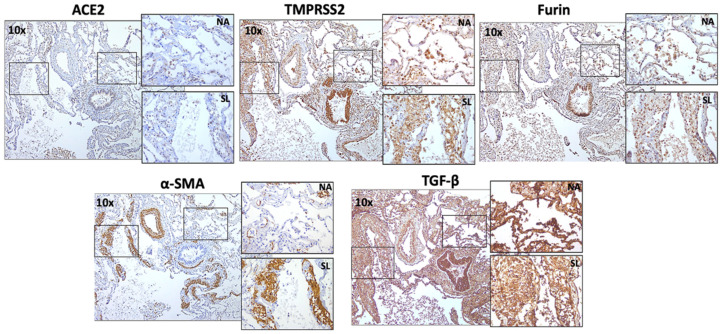
ACE2, TMPRSS2, Furin, myofibroblasts marker α-SMA, and growth factor TGF-β1 expression in “smooth muscle like” lesion and normal alveolar area in LAM patients. Representative images (10×) and inset images (40×) were taken using a bright field microscope. Each marker image was taken from the same subject in the same tissue area. NA—normal alveolar area; SL—“smooth muscle like” lesion.

In addition, in IPF patients, the fibrotic lesions showed less expression of TMPRSS2 and Furin, and moderate expression of ACE2 compared to the normal areas within the alveolar region (Figure 4, top panel). On the other hand, significant expression of TMPRSS2 and Furin in LAM patients and a more moderate ACE2 expression was observed in the “smooth muscle like” lesions than the normal alveolar area (Figure 5, top panel).

### 3.2. Descriptive Analysis of IPF and LAM Lung Tissue for α-SMA and TGF-β1

Additionally, growth factor TGF-β1 and myofibroblast markers α-SMA were variably expressed in tissue areas with the COVID-19 marker expression in fibrotic and non-fibrotic areas of IPF lungs (Figure 4, bottom panel) and “smooth muscle like” lesions and normal alveolar areas of LAM lungs (Figure 5, bottom panel). In IPF, TGF-β1 was more prominent in non-fibrotic alveolar areas than in fibrotic areas, whereas α-SMA expression remained unchanged. However, in LAM, increase in α-SMA and TGF-β1 was observed across the tissue, especially in the “smooth muscle like” lesions. The presence of TGF-β1 and COVID-19 biomarker expression in similar areas indicate their possible co-expression in LAM patients.

### 3.3. Correlation of ACE2 Expression and Smoking History in IPF Patients

**Figure 6:**
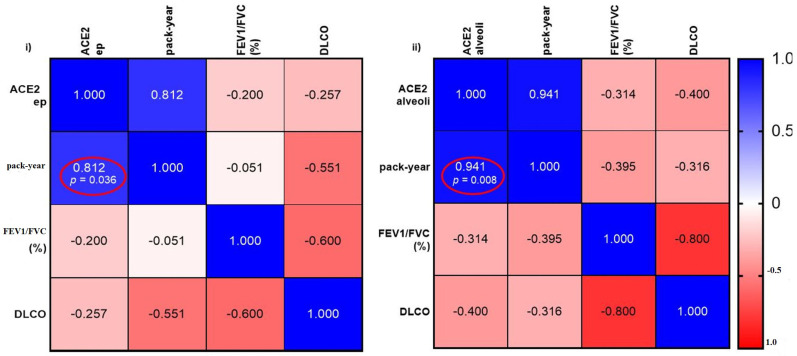
(**i**) Correlation between smoking history and ACE2 expression on SA epithelium (ep). (**ii**) Correlation between smoking history and ACE2 expression in alveolar areas in IPF patients. Significance is considered *p* < 0.05.

Compared to non-smokers, smokers are more vulnerable to respiratory infections [27]. IPF patients with a smoking history thus conjure a higher chance of susceptibility to microbial infections [28]. In patients with IPF, we found a significant positive correlation between smoking pack-years and ACE2 expression in the small airway epithelium (r = 0.812, *p* < 0.05) and alveoli (r = 0.941, *p* < 0.01; Figure 6i,ii. However, we found no correlation between smoking pack-years with TMPRSS2 or Furin expression. We also found no correlation between age and ACE2, TMPRSS2 or Furin expression in both IPF and LAM patients (data are not shown in the figure).

## 4. Discussion

This study, to our knowledge, is the first study that compares the ACE2, TMPRSS2, and Furin in resected lung tissue from IPF, LAM, and HC. We identified here that ACE2, TMPRSS2, and Furin are highly expressed on small airway epithelium and alveolar region in both IPF and LAM patients, especially high in IPF, which indicates that LAM and IPF patients could be more vulnerable to COVID-19 infections, and this could further exaggerate due to smoking history and comorbidities. Our findings provide the first histopathological evidence supporting the recent observational study from Lee et al. [29] The study reported that patients with ILD had higher susceptibility to COVID-19 and had more severe clinical outcomes from COVID-19, especially patients with IPF [29]. However, the reason for this increased risk for COVID-19 in IPF patients was not provided at a tissue level. Our current study brings novel mechanistic insights explaining why IPF and other ILD patients might be at risk of developing severe COVID-19. Clinical studies have also pointed out post-COVID-19 fibrotic changes and interstitial thickening at chest CT with a 6-month follow-up in survivors of COVID-19 without pre-existing ILD patients [30]. The ACE2, TMPRSS2, and Furin could be important biomarkers to provide insights into pre-COVID-19 and post-COVID-19 management. Our current study also shows increased expression of α-SMA+ myofibroblasts and variable expression of TGF-β1 in similar areas in both IPF and LAM tissue. Growth factors secreted by the myofibroblasts could further enhance the risk of COVID-19 infections and post-COVID-19 interstitial pulmonary fibrosis. Current results also match our previous findings with increased ACE2 expression in smokers and COPD [5,7,31], which conclusively indicates that an increased expression of ACE2, TMPRSS2, and Furin makes these patients extremely vulnerable to SARS-CoV-2 infections.

Current clinical evidence shows that IPF patients who were COVID-19 positive, developed severe fatal complications due to inflammation and lung injury, increasing their mortality from the virus-induced hyperinflammation [13]. This increased vulnerability to SARS-Cov-2 puts extra onus on IPF patients to take necessary precautions. Like IPF, LAM patients also suffer from progressive respiratory failure with a severe reduction in lung function. In the current study, LAM was the primary diagnosis in this patient cohort, however, some of these patients also had associated respiratory diseases, such as asthma, COPD, and pneumonia, which may have an add-on effect on ACE2, Furin and TMPRSS2 expression [3]. Our study complements Tang et al. [32] indicating an increase in COVID-19 module score, based on the expression of 332 human genes that interacted with SARS-CoV-2 in LAM pneumocytes, which was associated with positive expression for ACE2 and TMPRSS2. Here we further illustrate the in situ protein co-expression for ACE2, TMPRSS2, and Furin in LAM patients compared with IPF and healthy controls (Figure 1 and Figure 2), which has not been reported before. It is crucial to understand the signaling mechanisms involved in the upregulation of ACE2, TMPRSS2, and Furin in patients with ILDs.

ACE2 is the first step for the virus to attach to the host cell membrane [24,33]. Cofactor facilitating SARS-CoV-2 infectivity such as TMPRSS2 cleaves the spike (S) protein of SARS-CoV-2 and facilitates the fusion of SARS-CoV-2 and cellular membranes [24,25], thus a critical factor enabling cellular infection [34]. Furin is another potential protease to facilitate viral infections. It is highly expressed in the lung and implicated in viral infections on the respiratory tract by activating the surface glycoprotein, cleaving viral envelope glycoproteins, thereby enhancing viral fusion with host cell membranes [26]. In the current study, we observed increased positive expression of TMPRSS2 and Furin in the small airway epithelium and alveolar areas, which are majorly localized in cytoplasm and nuclei in both IPF and LAM sections, compared to HC (Figure 1).

On the other hand, LAM sections showed relatively lower expression of ACE2 and other co-factors in the alveolar epithelium than IPF. This reduction might be due to the established role of estrogens in downregulating ACE2 expression, as LAM is almost specific to females [35]. But interestingly no decrease in the small airway epithelium expression for ACE2, TMPRSS2, and Furin compared to IPF was seen. The fusion mechanisms of the SARS-CoV-2 are still under investigation. However, in our recent study, we observed increased early endosome antigen 1 (EEA1), late endosomes such as RAB7, cathepsin L, and lysosomal associated membrane protein (LAMP-1) in lung-resected tissue from smokers and patients with COPD [7].

Interestingly, we previously noted that the elevated endosomal protein markers expression and ACE2 expression were concomitant in small airways, type II pneumocytes and alveolar macrophages in smokers and COPD patients. These findings suggest that the endocytosis process is elevated in smokers and patients with chronic respiratory diseases, which means endocytic machinery could also play a critical role in assisting with the virus replication and release. In addition, due to smoking history with IPF in our cohort, we believe that endocytic vacuoles could also be active in this disease pathology, but this warrants further work.

There are limitations to the study. First, the sample size was small; however, the stats were robust. Therefore, we believe it is unlikely to be a type-2 error. Second, LAM patients have other respiratory comorbidities and received various treatments, such as Sirolimus, Armour Thyroid, Oxybutynin, Rapmine, Lyrica, and Pramipexole. These comorbidities and treatments may affect LAM patients’ ACE2, TMPRSS2, and Furin expression. Further studies are needed to investigate whether the comorbidities and treatments affect these markers’ expression [36,37,38,39]. Third, respiratory physiological data are unavailable in the current LAM cohort. It has limited us to correlate expression of markers and lung function parameters. Finally, both IPF and LAM patients were in severe disease conditions. We will further explore whether the severity of the disease and treatments would affect these biomarkers’ expression and if these changes are any different to early disease.

## 5. Conclusions

This is the first study to our knowledge that compares the ACE2, TMPRSS2, and Furin in resected lung tissue in IPF and LAM. We identified that ACE2, TMPRSS2, and Furin are highly expressed on small airway epithelium and alveolar regions in IPF and LAM patients. Notably, we observed the increased expression of these markers in type II pneumocytes and alveolar macrophages in IPF patients, indicating that LAM and IPF patients could be more vulnerable to COVID-19 infections, which could further exaggerate due to smoking history and comorbidities. It also indicates that more than one cell type is involved in these markers. Growth factors such as TGF-β1 could also be playing an essential role in upregulating these COVID-19-related biomarkers, further exaggerating fibrotic pathology; however, further work is needed to illustrate such changes. Other environmental factors such as air pollution and the use of electronic cigarettes could also be involved but further work is required in this space [40,41,42,43].

## Figures and Tables

**Table 1:** Subject demographics.

Groups	HC	IPF	LAM
**Subject number**	12	6	6
**Gender (F/M)**	6/6	3/3	6/0
**Years of age** **(median, range)**	42.5, 19–63	62, 56–70	55.5, 45–65
**Smoking status** **(Never-smoker/Ex-smoker)**	12/0	0/6	5/1
**FEV1/FVC% Post BD** **(mean ± SD)**	NA	89.32 ± 3.86	NA
**DLCO%** **(mean ± SD)**	NA	25.33 ± 12.3	NA
**Comorbidity**	NA	NA	Asthma, COPD, pneumonia, fibromyalgia, emphysema, arthritis
**Treatment**	NA	NA	Sirolimus, Armour Thyroid, Oxybutynin, Rapmine, Advair, Lyrica, Pramipexole

Abbreviations: HC—healthy controls; IPF—idiopathic pulmonary fibrosis; LAM—lymphangioleiomyomatosis; FEV1/FVC% Post BD—forced expiration/forced vital capacity% post bronchodilator; FVC% pred—forced vital capacity% predicted; DLCO%—Diffusing capacity for carbon monoxide%; COPD—chronic obstructive pulmonary disease; NA—not available; SD—standard deviation.

## Data Availability

All data are available upon request from the corresponding author.
